# Bloodstream infections in patients living with HIV in the modern cART era

**DOI:** 10.1038/s41598-019-41829-3

**Published:** 2019-04-01

**Authors:** L. Taramasso, F. Liggieri, G. Cenderello, F. Bovis, B. Giannini, A. Mesini, M. Giacomini, G. Cassola, C. Viscoli, A. Di Biagio

**Affiliations:** 1 University of Genova (DISSAL), Infectious Diseases Clinic, Policlinico Hospital San Martino, Genova, Italy; 2 Infectious Diseases Unit, Department of Internal Medicine, Fondazione IRCCS Ca’ Granda, Ospedale Maggiore Policlinico, Milan, Italy; 3 Infectious Diseases Unit, EO Ospedali Galliera, Genova, Italy; 4 Biostatistics Unit, Department of Health Sciences, University of Genoa, Genoa, Italy; 5 Department of Informatics, Bioengineering, Robotics and System Engineering (DIBRIS), University of Genova, Genova, Italy; 6 Infectious Diseases Clinic, Policlinico Hospital San Martino, Genova, Italy

## Abstract

Retrospective multicentre study aiming at analysing the etiology, characteristics and outcome of bloodstream infections (BSI) in people living with HIV (PLWHIV) in an era of modern antiretroviral therapy. Between 2008 and 2015, 79 PLWHIV had at least 1 BSI, for a total of 119 pathogens isolated. Patients were mainly male (72.1%), previous intravenous drug users (55.7%), co-infected with HCV or HBV (58.2%) and in CDC stage C (60.8%). Gram-positive (G+) pathogens caused 44.5% of BSI, followed by Gram-negative (G−), 40.3%, fungi, 10.9%, and mycobacteria, 4.2%. *Candida* spp. and coagulase-negative staphylococci were the most frequent pathogens found in nosocomial BSI (17% each), while *E.coli* was prevalent in community-acquired BSI (25%). At the last available follow-up, (mean 3.2 ± 2.7 years) the overall crude mortality was 40.5%. Factors associated with mortality in the final multivariate analysis were older age, (p = 0.02; HR 3.8, 95%CI 1.2–11.7) CDC stage C (p = 0.02; HR 3.3, 95%CI 1.2–9.1), malignancies, (p = 0.004; HR 3.2, 95%CI 1.4–7.0) and end stage liver disease (p = 0.006; HR 3.4, 95%CI 1.4–8.0). In conclusion, the study found high mortality following BSI in PLWHIV. Older age, neoplastic comorbidities, end stage liver disease and advanced HIV stage were the main factors correlated to mortality.

## Introduction

Due to the availability of modern combined antiretroviral therapy (cART), patients living with HIV (PLWHIV) have experienced a reduction in overall mortality and incidence of AIDS-defining conditions^1,2^. The natural history of HIV infection has changed, evolving from a disease with a bad short-term prognosis to a long-term chronic infection, forcing physicians to focus on new clinical problems, such as aging, kidney disease, osteoporosis, diabetes, cardiovascular diseases and the effects of long-term drug exposure. Indeed, late complications in otherwise responding HIV-infected patients exist and include, among others, neoplastic diseases requiring chemotherapy, putting these patients at risk of opportunistic infections similar to those encountered in the non-HIV positive population with the same cancer. In the meantime, in some countries, including Italy, up to 29% of the patients arrive late at a HIV diagnosis, with CD4+ T-cell (CD4+) count <200/mmc or with an AIDS-defining condition (infection and/or cancer) already present at the moment of diagnosis^3^. In this patient population, the immunological recovery can be challenging^4,5^, and they may require long hospitalization for the combined treatment of the HIV infection, AIDS-related infections and sometimes neoplastic diseases^6^. At this point, they are at risk of developing classic healthcare-associated infections, such as bacteremia, which further complicate an already difficult situation. Indeed, non-AIDS defining bacterial infections are among the main causes of hospital admissions nowadays^7–10^.

The aim of the present study is to analyze the etiology, clinical characteristics and outcome of bloodstream infections (BSI) in PLWHIV in an era of cART in a country with a high rate of late-presenters.

## Results

**Figure 1:**
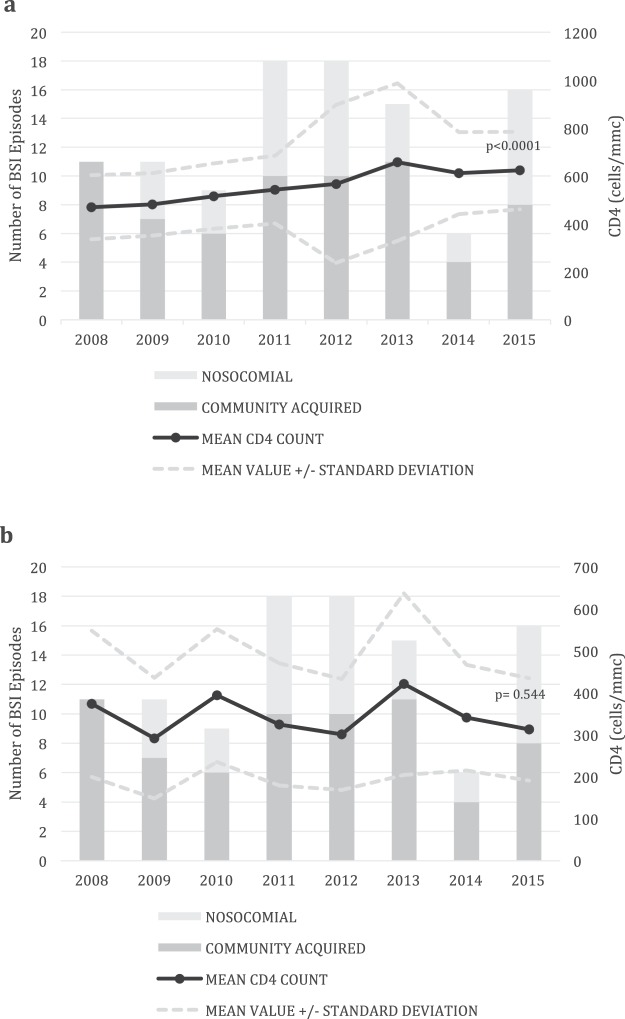
Number of community acquired and nosocomial episodes of bloodstream infections (BSI) and CD4+ T-lymphocytes (CD4) count in the whole study population (**a**) and in patients newly diagnosed with HIV (**b**) in the years of the study. P values in the graph are referred to the CD4 trend across the study period.

Throughout the study period, 2379 PLWHIV were followed in the 2 participating centers with 2281 hospitalizations. A total of 79/2379 (3.3%) patients had at least 1 BSI, for a total of 104 episodes (67, 64.4%, community acquired and 37, 35.5%, hospital-acquired, Fig. 1) and 119 isolated pathogens. BSI accounted for or complicated a total of 93 of 2281 hospitalizations (4.0%), with a median duration of hospital stay of 17 (10–36) and 59 (31.5–100.5) days in community-acquired and nosocomial episodes, respectively (p < 0.0001).

The majority of PLWHIV with BSI were male (72.1%), previous intravenous drug users (55.7%), co-infected with HCV or HBV (58.2%) and in CDC stage C (60.8%). General characteristics of the study population are given in Table 1. The mean follow-up period after the first BSI episode was 3.2 ± 2.7 years.

**Table 1:** General characteristics of the study population at time of first bloodstream infection (79 patients).

Age, median (1^st^–3^rd^ quartile)	47 (20–78)
Gender (Male), n (%)	57 (72.1)
HBV/HCV coinfection, n (%)	46 (58.2)
Last CD4 < 100 N/mmc, n (%)	24 (30.4)
Last CD4 < 200 N/mmc, n (%)	45 (56.9)
Late Presenter, n (%)	19 (24.0)
On co-trimoxazole prophylaxis, n (%)	21 (26.6)
***On antiretroviral treatment, n (%)***	52 (65.8)
***- NRTI*** **+** ***NNRTI***	6 (11.5)
***- NRTI*** **+** ***PI***	31 (59.6)
***- NRTI*** **+** ***INSTI***	9 (17.4)
***- Other regimens***	6 (11.5)
HIV-RNA ≥ 200 copies/ml, n (%)	48 (60.7)
Detectable HIV-RNA despite antiretroviral treatment, n (%)	21 (26.6)
Patients with at least one N-BSI, n (%)	27 (34.2)
Patients with at least one CA-BSI, n (%)	57 (72.1)
Patients with both N- and CA-BSI, n (%)	4 (5.1)
***Patients with more than one episode of BSI, n (%)***	14 (17.7)
*CDC stage B, n (%)*	28 (35.4)
*CDC stage C, n (%)*	48 (60.8)
***Route of transmission***	
*Vertical, n (%)*	4 (5.1)
*Drug addiction, n (%)*	44 (55.7)
*Sexual, n (%)*	31 (39.2)
***Comorbidities***	
*HBV/HCV co-infection, n (%)*	46 (58.2)
*- Cirrhosis, n (%)*	26 (32.9)
*- End stage liver disease, n (%)*	9 (11.4)
*- HCC, n (%)*	1 (1.3)
*Malignancies, n (%)*	14 (17.7)
*- Solid cancer, n (%)*	7 (8.9)
*- Lymphoma, n (%)*	6 (7.6)
*- Leukimia, n (%)*	1 (1.3)
*Psychiatric disorders, n (%)*	5 (6.3)
*Encephalopathies, n (%)*	14 (17.7)
*Diabetes, n (%)*	7 (8.9)
*Chronic kidney disease, n (%)*	9 (11.4)
*Current IVDU, n (%)*	23 (29.1)

NRTI: nucleoside reverse transcriptase inhibitors; NNRTI: non-nucleoside reverse transcriptase inhibitors; PI: protease inhibitors; INSTI: Integrase strand transfer inhibitors; N-BSI: nosocomial bloodstream infection; CA-BSI: community-acquired bloodstream infection; HCC: hepatocellular carcinoma; IVDU: intravenous drug use.

The median CD4+ count at first BSI was 175 (88–331.5) cells/μl as compared to 420 (252–610) in the general population of PLWHIV observed at the first visit to the 2 outpatient clinics during the study period (p < 0.0001). Among patients with at least 1 BSI, the proportion of late-presenters was 24.0% (19 of 79) as opposed to 10.4% (239 of 2304) in the group of patients without any BSI (p < 0.0001).

A total of 14 patients developed more than 1 BSI episode. They had similar characteristics as compared with patients with a single episode (i.e., age, sex, CDC stage, frequency of HBV and HCV co-infection and of late presentation, CD4+ count and nadir at the time of the first BSI).

Among the 119 isolated pathogens, there were 53 (44.5%) Gram-positive (G+) cocci, 48 (40.3%) Gram-negative (G−) rods, 13 (10.9%) fungi (9 candidemia and 4 cryptococcal infections) and 5 (4.2%) mycobacteria (2 *M. tuberculosis* complex and 3 *M. avium* complex). Candidemia was caused by *C. albicans* in 6/9 cases, *C. glabrata*, *C. tropicalis* and *C. parapsilosis* in 1/6 cases each. The median CD4+ T-cell count (cells/μl) was 210 (102–299.5) in patients who developed a BSI caused by G−, 132 (60–250) in G+, 77 (11.5–120) in mycobacteria and 62 (35–215) in fungal BSI, with a significant decreasing trend in the different groups of etiologies (p = 0.011). Parallelly, the frequency of late presenting in each group of etiologies was also different, higher in mycobacterial (80.0%) than in fungal (53.8%), G+ (24.5%) and G− (14.6) BSI, p < 0.0001.

In Table 2, all pathogens are classified, whether isolated in nosocomial or community-acquired episodes. As expected, *Candida* spp. and coagulase-negative staphylococci (CONs) were more often nosocomial in origin (8 of 9 each), while *E.coli* was prevalent in community-acquired BSI (18 episodes, 24.6%). With respect to community-acquired episodes, nosocomial BSI were more often observed in patients on antiretroviral treatment and with BSI caused by *Candida* spp. On the other side, hospitalization for an acute infectious disease or febrile syndrome was protective towards nosocomial episodes, compared to other causes of hospitalization (area under the curve of the model, AUC = 0.855, Table 3).

**Table 2:** Number, type and distribution of 119 BSI causative agents.

Pathogen	Total	C A	N
*Escherichia coli*	20	18	2
*Staphylococcus aureus*	14	8	6
Other *Streptococcus* spp	12	9	3
*Enterococcus* spp	11	6	5
*Candida* spp	9	1	8
CONs	9	1	8
*Pseudomonas* spp	9	4	5
*Klebsiella pneumoniae*	8	4	4
*Streptococcus pneumoniae*	5	5	0
*Mycobacteria* spp	5	5	0
*Cryptococcus neoformans*	4	3	1
*Salmonella* spp	2	2	0
*Serratia* spp	2	2	0
Other G−	7	3	4
Other G+	2	2	0
**Total**	**119**	**73**	**46**

CA: community acquired; N: nosocomial; CONs: Coagulase-Negative Staphylococci; G−: Gram negative; G+: Gram positive.

**Table 3:** Comparison of factors associated with nosocomial vs. community acquired BSI in 79 patients (119 infections).

Variable	Beta Estimate	95%CI	p-value	Multivariate Beta Estimate	Multivariate 95%CI	Multivariate p-value
Age < 47 years*	0.6	0.2–1.4	0.22			
CD4 < 200	1.0	0.4–3.9	0.97			
Decease at last follow up	0.8	0.3–1.9	0.56			
End stage liver disease	0.8	0.3–2.0	0.59			
G−	1.6	0.7–3.9	0.30			
G+	1.2	0.5–2.7	0.62			
Fungi	3.8	1.3–10.8	0.01			
*Candida* spp.	4.9	1.7–14.5	0.003	18.1	1.7–186.8	0.01
HIVRNA ≥ 200 cp/ml	1.5	0.5–4.3	0.41			
Active IVDU	1.7	0.6–4.5	0.28			
Late presentation	1.1	0.6–2.2	0.68			
Male gender	1.1	0.4–3.2	0.80			
On cART	3.3	1.3–8.0	0.009	4.1	1.3–13.1	0.02
N of previous infections	1.7	0.4–7.9	0.51			
Co-trimoxazole prophylaxis	1.1	0.4–3.3	0.82			
CDC stage C	0.9	0.3–2.2	0.75			
**Risk factor for HIV infection**						
IVDU	1.1	0.4–2.9	0.78			
Vertical transmission	1.2	0.2–6.7	0.85			
Sexual	0.8	0.3–2.2	0.73			
**Comorbidities**						
malignancies	1.3	0.5–2.9	0.59			
Chronic kidney disease	0.2	0.02–2.1	0.20			
HCV/HBV co-infection	1.2	0.5–3.1	0.68			
Cirrhosis	1.8	0.7–4.5	0.22			
Other comorbidities	0.9	0.3–2.4	0.78			
**Admitting dianosis**						
Sepsis	0.1263	0.02–0.5	0.005			
Acute infectious disease or fever	0.0929	0.03–0.2	<0.0001	0.06	0.02–0.2	<0.0001

Area under the curve of the multivariate model (AUC) = 0.855.

CI: confidence interval; N: number; cp: copies; G+: Gram positive; G−: Gram negative; cART: combined antiretroviral therapy; IVDU: intravenous drug use.

*dichotomized as per ROC.

In terms of antimicrobial susceptibility, no G− rod was resistant to carbapenems, while 8 out of 37 Enterobacteriaceae and 0 of 9 *Pseudomonas* spp. were ESBL-producers. Among G+ cocci, 7 out of 9 CONs (7/7 nosocomial) and 8 of 14 *S. aureus* strains (3/8 nosocomial) were resistant to methicillin, while 9 of 11 enterococci were resistant to ampicillin. All but one G+ (i.e., one nosocomial-acquired *E. faecium*) were susceptible to vancomycin. Two of 9 candidemias were due to fluconazole resistant strains (one *C. albicans* and one *C. glabrata)*.

The observational analysis showed that the crude mortality after the first BSI episode in the 79 patients was 6.3%; 8.9%, 21.5% and 27.8% at 2, 4, 24 and 48 weeks, while the overall mortality at the last available follow up was 40.5%, (32/79 patients), with a median time between infection and death of 231 days (48–438). Four factors were associated with mortality at multivariable analysis: age >43 years (p = 0.02; HR 3.8, 95%CI 1.2–11.7), CDC stage C (p = 0.02; HR 3.3, 95%CI 1.2–9.1), malignancies (p = 0.004; HR 3.2, 95%CI 1.4–7.0), and end stage liver disease (p = 0.006; HR 3.4, 95%CI 1.4–8.0) (Table 4).

**Table 4:** Univariate and multivariate predictors of event (mortality at the last follow-up) in 79 patients. The multivariate analysis was performed on 77 patients with fully available data.

	HR (95% CI) univariate	p-value	HR (95% CI) multivariate	p-value
Age	1.035 (1.003–1.068)	**0.03**		
Age > 43*	4.0 (1.4–11.5)	**0.009**	3.8 (1.2–11.7)	**0.02**
Gender (Male)	1.5 (0.7–3.5)	0.33		
HBV/HCV co-infection	1.8 (0.8–4.0)	0.13		
Last CD4 < 200 N/mmc	1.6 (0.8–3.2)	0.22		
HIVRNA ≥ 200 copies/ml	0.7 (0.3–1.4)	0.31		
Nr of previous infections	1.3 (1.0–1.6)	**0.02**		
CDC stage C	3.4 (1.4–8.3)	**0.008**	3.3 (1.2–9.1)	**0.02**
***Etiology***				
*- Fungi*	3.5 (1.3–9.1)	**0.01**		
*- Gram*+	0.8 (0.4–1.7)	0.67		
*- Gram*−	0.8 (0.4–1.7)	0.63		
*- Mycobacteria*	0.5 (0.1–3.9)	0.54		
***Route of transmission***				
*- Vertical*	1.3 (0.3–5.5)	0.72		
*- Drug addiction*	1.5 (0.7–3.1)	0.24		
*- Sexual*	0.6 (0.3–1.3)	0.18		
Nosocomial	1.4 (0.7–2.9)	0.33		
Resistance to Beta-lactams**	1.8 (0.8–4.1)	0.18		
Late Presentation	0.8 (0.3–2.1)	0.69		
***Comorbidities***				
*-HBV/HCV co-infection*	1.8 (0.8–4.0)	0.13		
* - Cirrhosis*	1.7 (0.8–3.3)	0.16		
* - End stage liver disease*	3.9 (1.8–8.5)	**0.0006**	3.4 (1.4–8.0)	**0.006**
*- Malignancy*	3.3 (1.6–6.8)	**0.001**	3.2(1.4–7.0)	**0.004**
*- Chronic kidney disease*	2.3 (0.9–5.6)	0.07		
*- Other comorbidities*	1.3 (0.6–2.6)	0.48		
Current IVDU	1.4 (0.7–3.0)	0.37		
Cotrimoxazole prophylaxis	2.1 (1.1–4.3)	**0.03**		
Not on cART	1	0.79		
On cART, HIV-RNA ≥ 200 copies/ml	1.0 (0.4–2.6)			
On cART, HIV-RNA < 200 copies/ml	1.3 (0.5–3.0)			
Admission diagnosis of infectious disease or fever	0.5 (0.3–1.1)	0.09		
Admission diagnosis of Sepsis	1.9 (0.4–7.9	0.40		

*Dichotomized as per ROC.

**Calculated for 70 patients with bacterial bloodstream infections. Results were interpreted in accordance with the European Committee on Antimicrobial Susceptibility Testing (EUCAST) clinical breakpoints. Staphylococci were considered resistant if they were not susceptible to methicillin; Streptococci were considered resistant if they were not susceptible to amoxicillin; Enterococci were considered resistant if they were not susceptible to ampicillin; Gram negative bacteria were considered resistant if they were not susceptible to third generation cephalosporins or piperacillin/tazobactam.

HR: Hazard Ratio; IVDU: intravenous drug use; cART: combined antiretroviral therapy.

Admission diagnoses were referred to last hospitalization for patients with multiple episodes.

Other comorbidities comprise: any encephalopathies (14 patients), diabetes (7 patients), psychiatric disorders (5 patients) and Chronic Obstructive Pulmonary Disease (3 patients).

Malignancies comprise solid cancers and hematological malignancies.

## Discussion

In the present study, we analyzed the etiology and outcome of BSI in PLWHIV in the years 2008 to 2015 at two Infectious Disease Centers in Northern Italy. Despite the fact that the study has been conducted in the context of a modern cART era, only 66% of the patients were actually on antiretroviral treatment, and a high number of late presenters (24%) and patients in advanced stage of disease (61% in CDC stage C) were still present in our series. The patients who developed BSI had lower CD4+ counts and higher frequency of late presenters when compared to the global population of PLWHIV without BSI, confirming the importance of immune recovery for also preventing bacterial infections other than those commonly codified as AIDS-defining^11^. Indeed, we found a quite high crude mortality rate in the first weeks after BSI, with a continuous increasing trend in the months following the episode, arriving to 40% after a median time of 231 days. This high mortality rate likely reflects the severity of the underlying diseases of patients who develop BSI and the high frequency of comorbidities in PLWHIV, possibly favored by a somewhat inadequate immunological reconstitution and by chronic immune activation^12^. This is shown by the fact that patients with BSI had lower CD4+ counts than the general population of PLWHIV and that, among them, mortality was higher in those with older age, more advanced CDC stage and comorbidities such as malignancies or end stage liver disease. Surprisingly, we did not find a reduced HR for mortality in patients on cART, whether they had HIV-RNA < 200 copies/ml or not. However, cART is expected to diminish the risk of comorbidities found to be associated with higher mortality in our series, such as neoplastic diseases or AIDS-defining illnesses^13^, so that it is possible that the lack of association between cART use and reduced mortality might be simply due to the small sample size.

On the other hand, the high frequency of nosocomial episodes found suggests how BSI can also complicate the clinical course in otherwise responding HIV-infected patients, as these episodes were not linked to the CDC stage of disease but on the contrary more frequent in patients taking antiretroviral treatment who were mainly hospitalized for non-infectious illnesses.

The use of a modern cART might however have played a role in the general study population, as we found that the epidemiology of pathogens causing BSI changed with respect to previous data^14–24^. In fact, we found a high prevalence of G− as the cause of community-acquired BSI, with *E. coli* as the main causative agent overall. Even if an increasing prevalence of this pathogen had already been signaled in PLWHIV^15^, in our knowledge, it was the principal etiology of all BSI only in one previous study^24,25^. This change may be linked to easier and earlier access to cART in comparison with the past, with consequent decreasing frequency of “AIDS-defining BSI” and increase of other common causes of BSI, shared with HIV-negative patients^26^.

Less data is available on the etiology of hospital-acquired BSI in PLWHIV^14,16,27–29^. Candidemia had previously been reported as an emergent cause of BSI in PLWHIV^25^, but its burden seemed still higher in our work, where it was the first cause of nosocomial BSI and significantly correlated with the nosocomial occurrence of the infection. A possible interpretation is that the better survival of PLWHIV is burdened by longer and more frequent hospitalizations, and this constitutes a well-known risk factor for the development of candidemia^30^. Indeed, nosocomial BSI in PLWHIV has increased in last years^14,31^ and constitutes about 35% of the episodes in our work.

Finally, we also assessed the resistance profile of principal G−, G+ and *Candida* spp. isolated from blood cultures. Despite the fact that resistance to carbapenems is a major concern in Italy, even with increasing rates in last years^32^, it did not have a major impact in PLWHIV during the study period, as no G− harbored carbapenem-resistant rods. Moreover, the majority of Enterobacteriaceae were susceptible to third generation cephalosporins, also if some ESBL producer strains were found. Numbers are too low to draw any conclusions, but on the basis of the descriptive analysis of our population, we can only say that the rising resistance reported in other contexts^33^ seem not to spare PLWHIV. The same is true also for G+. In fact, we found a quite high proportion of community acquired methicillin resistant *S. aureus*, in accordance with the literature data^34^, while among CONs methicillin resistance was confined to nosocomial episodes. A single vancomycin resistant *E. faecium* was isolated, while all other G+ conserved vancomycin susceptibility.

The study has several limitations. A major limit is that we did not have a control group, so we could not demonstrate that the mortality we found, although unusually high, was significantly different from the mortality in PLWHIV without BSI with similar clinical conditions. Moreover, the retrospective design did not permit the analysis of all underlying conditions and concomitant therapies that may have influenced both the primary and secondary outcomes of the study. For the same reason, we cannot exclude an underestimation of the real number of BSI, as it is possible that in some cases either the blood cultures were not promptly collected or early antimicrobial treatment gave negative results before hospital admission or blood collection. In addition, changes in infection-control practices or in the distribution of pathogens over time in the participating centers may have played a role in the epidemiologic framework described in the article that appeared different from those previously described in other similar studies^13–23^. Finally, the relatively low incidence of BSI in PLWHIV did not permit the analysis of a large amount of data, and fortuity may have played a role in the definition of some variables or in the lack of significance of others.

With these limitations, our study highlights new emerging features of BSI in PLWHIV in the recent cART era. BSI share the etiologies of both community-acquired and nosocomial episodes with HIV-negative patients but are still marked by high mortality, correlated not only to an advanced HIV stage but also to other major comorbidities. In the future, we expect that the scenario will change again as in the present study the history of intravenous drug use, now a declining risk factor, and HCV co-infection, now present only in a minority of new HIV diagnosis, are still highly represented^35,36^. Moreover, there are still numerous patients with low CD4+ counts and with advanced diseases. Both hopefully will reduce in the next years due to new and earlier cART strategies. Multicenter and prospective studies in the future might better define BSI epidemiological changes, risk factors and outcomes in the continually evolving population of PLWHIV.

## Methods

This is a retrospective study conducted in the period 2008–2015 at two Infectious Diseases Units in the city of Genoa (Italy, Liguria Region), the Ospedale Policlinico San Martino and the Ente Ospedaliero Ospedali Galliera. The 2 units follow slightly more than 2000 HIV-positive patients in a Region with an estimated overall number of about 3000 HIV-infected patients in treatment or follow up.

Data about all HIV-infected patients >18 years followed at the two centers were retrieved from the Database of the Liguria HIV Network (RLH-DB): (www.reteligureHIV.it). The Liguria HIV Network is a locally developed online platform that supports the direct connection between medical and laboratory records of HIV-infected patients, allowing the automatic and prospective transfer of anonymized data^37,38^. Each patient has an identification code, which is registered in RLH-DB at the engagement (first ambulatory visit for outpatients or first day of hospital stay for inpatients). Safety and precision are granted by the approved use of hospital anonymized codes. The use of RLH-DB was approved by the Ligurian Ethical Committee. At the moment of registration in RHL-DB, patients sign an informed consent form in which they declare if they agree to use their clinical data, in anonymous form, for scientific purposes. The study has been performed in accordance with the ethical standards laid down in the 1964 Declaration of Helsinki and its later amendments and in accordance with Italian national laws.

All patients registered in RLH-DB who gave written consent to study participation were considered eligible. The BSI episodes were extrapolated by revision of all positive blood cultures registered in the online platform. The number of hospitalizations in PLWHIV was obtained by reviewing discharge diagnostic codes of all patients hospitalized during the study period, on the basis of the International Classification of Diseases (ICD-9).

BSI was defined as at least one positive blood culture for fungi or bacteria in the setting of a compatible clinical disease. For BSI due to the same pathogen (microbial or fungal) at least 30 days between subsequent episodes were required to define separate BSI episodes. For BSI due to mycobacteria, multiple positive blood cultures, in the same patient, were considered as one single episode. For blood cultures positive for more than one pathogen within 48 hours, a single, polimicrobial, episode was considered. For common skin contaminants, like coagulase-negative staphylococci, *Corynebacterium*, *Peptostreptococcus, Bacillus* and *Propionibacterium* species, at least 2 positive blood cultures were required. For cryptococcosis only cases with positive blood cultures were included. For each patient the following information were collected at baseline (at the time of RLH-DB registration) or at time of BSI. Baseline information included gender, HBV co-infection (defined by HBsAg positivity), HCV co-infection (defined by qualitative HCV-RNA positivity), CDC stage (www.CDC.org), CD4+ count nadir. Information at time of each BSI included age, CD4+ count, detectable or undetectable (<200 copies/ml) plasma HIV-RNA, site of acquisition (nosocomial if first positive blood culture after at least 48 hours of admission, community-acquired if positive blood culture within the first 48 hours of hospitalization or in case of mycobacterial etiology), etiology of BSI, susceptibility pattern of the isolated pathogen (for *Candida* spp. only fluconazole susceptibility). Isolates were identified with the Vitek 2 system (bioMérieux, Marcy l’Etoile, France) and/or by matrix-assisted laser desorption ionization-time-of-flight (MALDI-TOF) mass spectrometry (MALDI Biotyper, Bruker Daltonics, Leipzig, Germany, or VitekMS, bioMérieux). The *in vitro* susceptibility of the isolates was assessed with the Vitek 2 system (bioMérieux). Results were interpreted in accordance with the European Committee on Antimicrobial Susceptibility Testing (EUCAST) clinical breakpoints. Major comorbidities and primary causes of hospitalizations were extrapolated by the RLH-DB data and the discharge diagnostic codes ICD-9 at each hospitalization. Patients who had CD4+ count <350 cells/µl at the moment of the diagnosis of HIV infection were defined as late presenters^39^. Number of BSI per patient was also collected, as well as the total number of hospitalizations in the overall cohort and the number of hospitalizations due to or complicated by a BSI. The outcome (survival) was evaluated at 2, 4, 24, 48 weeks after the episode and thereafter till the last available follow-up. The computerized systems for patient registration used by the participating centers, allow information on the deaths on the entire nation that are notified and updated in real time thanks to connection with the national registry office.

### Statistical analysis

Quantitative data are presented as medians (1st and 3rd quartile) or means (±standard deviation) and categorical data as absolute numbers and percentages. Quantitative data were compared by Mann-Whitney U test, or, when more than two groups were compared, by Kruskal-Wallis test. Categorical variables were compared by chi-square test. Cox regression model was used to assess the impact of the above mentioned variables on survival. For the analysis of survival only the etiology of the last episode was considered. The impact of the same variables on site of BSI acquisition (community versus hospital acquired) was investigated using a generalized linear model, in which BSI episodes within the same subject were assumed as correlated to each other. Age was dichotomized according to the best threshold obtained from the receiver operating characteristic (ROC) curve analysis^40^. Only factors significantly associated with the outcome at univariate analysis were included in a multivariate model with a stepwise procedure. P-values < 0.05 were considered statistically significant. All analyses were carried out using the SAS software version 9.3 (Institute Inc., Cary, NC, USA).

## Data Availability

The dataset used and analyzed during the current study is available from the corresponding author on reasonable request.
